# Force-sensing drill-through detection and automatic stopping in a porcine distal humerus model simulating pediatric supracondylar pinning

**DOI:** 10.3389/fped.2026.1780738

**Published:** 2026-02-19

**Authors:** Kunzhi Zhu, Juxiang Huang, Gang Chen, Yuan Pan, Chaoran Hu, Yingying Deng, Yunfeng Xu, Lianyang Lin, Chao Feng

**Affiliations:** 1 Guizhou Medical University, Guiyang, China; 2 Beijing Jishuitan Hospital Guizhou Hospital, Guiyang, China; 3 Beijing University of Posts and Telecommunications, Beijing, China; 4 Beijing Jishuitan Hospital, Capital Medical University, Beijing, China

**Keywords:** automatic drill stopping, force sensing, kirschner wire fixation, overdrill control, pediatric supracondylar humeral fracture, robotic assistance

## Abstract

**Background:**

Supracondylar humeral fractures are the most common elbow fractures in children and are often treated with closed reduction and percutaneous Kirschner wire (K-wire) fixation. After far-cortex breach, delayed stopping can cause overdrilling and jeopardize adjacent neurovascular structures. We evaluated a force-sensing drill-through stopping system in a porcine humerus model and assessed the effects of feed rate and spindle speed on overdrill depth.

**Methods:**

Porcine distal humeri were drilled with a 2.0-mm K-wire introduced through the lateral condyle at ∼60° to the humeral longitudinal axis. A si*x*-axis force/torque sensor measured axial force; a transient force drop triggered an automatic stop command. Overdrill depth was the distance the K-wire tip advanced beyond the outer surface of the far cortex. Parameter tests compared feed rates (0.5/1.0/1.5 mm·s^−^¹ at 1,200 r min^−^¹) and spindle speeds (900/1,200/1,500 r min^−^¹ at 1.0 mm s^−^¹). Robotic vs. manual drilling was evaluated in paired tests at adjacent, non-interfering sites; manual drilling was performed by a senior pediatric orthopedic surgeon using tactile feedback. Statistical analysis used repeated-measures one-way ANOVA with Geisser–Greenhouse correction and Tukey *post hoc* tests, and paired t-tests (*α* = 0.05).

**Results:**

Feed rate significantly affected overdrill depth (Geisser–Greenhouse corrected, *p* = 0.016); 1.5 mm·s^−^¹ produced greater overdrill depth than 0.5 mm·s^−^¹ (Δ = 0.252 mm, adjusted *p* = 0.0456). Spindle speed had no significant effect (*p* = 0.900). In paired comparisons, the robotic system reduced overdrilling from 6.60 ± 1.53 mm (manual) to 0.87 ± 0.12 mm (robotic) (mean paired difference 5.73 mm, 95% CI 4.67–6.80; *p* < 0.0001), an 86.8% reduction.

**Conclusions:**

The force-sensing drill-through stopping system limited overdrill depth to approximately 1 mm in a porcine humerus model. Within the tested range of 900–1,500 r·min^−^¹ and 0.5–1.5 mm·s^−^¹, higher feed rates produced a modest increase in overdrilling whereas spindle speed had no significant effect. Compared with manual drilling, the system substantially reduced overdrill depth (≈1 mm vs. 6.6 mm), suggesting potential safety advantages during percutaneous pinning by limiting overdrilling and thereby increasing the safety margin after far-cortex breakthrough. Clinical studies are warranted to determine whether this translates into fewer neurovascular complications.

## 1 Introduction

Supracondylar humeral fractures are the most common type of elbow fracture in children, accounting for approximately 50%–60% of pediatric elbow fractures (1). For displaced Gartland type II and III fractures, closed reduction followed by percutaneous crossed Kirschner wire (K-wire) fixation is the standard treatment to maintain alignment and facilitate healing (2). However, the distal humerus in children has a dense cortical structure with closely adjacent neurovascular anatomy, which creates a narrow operative corridor and poses significant technical demands. Inadequate reduction may lead to residual varus or valgus deformity, while repeated attempts during pinning may increase soft-tissue damage and neurovascular complications (3).

During percutaneous pinning, the surgeon must stop wire advancement precisely at the moment of far cortex breakthrough to prevent excessive K-wire protrusion and potential neurovascular injury (4). Traditional manual drilling relies heavily on the surgeon's tactile feedback and experience. Human reaction time and drill inertia often result in overdrilling once the far cortex is breached, making it difficult to stop the wire at the “just-through-the-cortex” safety boundary. Previous studies have reported overshoot of the far cortex ranging from several millimeters to more than one centimeter, with the magnitude influenced by surgical experience (5, 6). In the pediatric elbow—where anatomical spaces are narrow and neurovascular structures are densely clustered—even millimeter-scale overdrilling may cause significant injury (7, 8).

To mitigate these risks, force-sensing closed-loop stopping strategies have been proposed in recent years. By continuously monitoring axial force or torque, these systems detect the characteristic sudden drop associated with cortical breakthrough. Compared with manual trigger release, automatic shutdown can markedly reduce stopping delay and, by interrupting feed motion immediately at breakthrough, enable rapid and precise cessation of drilling (9, 10). Such approaches have the potential to confine post-breakthrough overdrilling to approximately 1 mm, which may increase the mechanical safety margin after far-cortex breakthrough in this model.

Building on this concept, the present study used a porcine distal humerus model with two aims: (1) to evaluate the influence of feed rate and spindle speed on overdrill control by a force-sensing automatic stopping system; and (2) to directly compare this system with conventional manual drilling under identical experimental conditions. We hypothesized that the system would demonstrate greater consistency and stability across parameter settings and, relative to manual operation, would significantly reduce overdrilling at the moment of far cortex breakthrough.

## 2 Materials and methods

### 2.1 Study design and primary outcome

A standardized *ex vivo* bone model was used in a paired controlled design with repeated measures. The primary outcome was post-penetration overdrill depth (mm), defined as the linear distance by which the K-wire tip advanced beyond the outer surface of the far cortex after breakthrough. Within the robotic drill-through stopping system, we evaluated the effects of feed rate and spindle speed on overdrilling and assessed the system's consistency and robustness under repeated measurements. In addition, paired comparisons were performed at adjacent, non-interfering sites on the same bone to compare overdrill depth between the robotic system and manual drilling performed by a senior pediatric orthopedic surgeon, who stopped wire advancement based on tactile feedback. Hole allocation and drilling order were randomized using a computer-generated sequence. Overdrill measurements were performed by assessors blinded to group assignment and parameter level. Outcome assessment and blinding. Overdrill-depth measurements were performed by investigators not involved in drilling execution. After drilling, each hole was assigned a randomized identifier for outcome recording. Overdrill-depth results were not disclosed to the surgeon after individual manual holes; values were unmasked only after completion of all paired blocks and final data entry to minimize potential learning or performance drift. Operator blinding was not feasible because the robotic and handheld procedures are inherently different.

### 2.2 Specimens and preparation

Because of ethical and availability constraints, pediatric distal humeral specimens could not be obtained. Following previous experimental work, distal porcine humeri were therefore used as a surrogate model (11). Porcine bone has been shown to resemble human bone at both anatomical and histological levels, particularly with respect to the microstructure of trabecular and cortical bone and patterns of mineralization and remodeling (12). Distal humeri were harvested from freshly slaughtered pigs (age 6–8 months, body weight 90–100 kg) at a local abattoir. All soft tissues and periosteum were removed, and specimens were kept moist throughout preparation and testing with saline-soaked gauze at room temperature (24 °C).

Each bone was rigidly fixed in a custom drilling platform using mechanical clamps. To minimize mechanical interaction between adjacent drill holes, a regular grid was drawn on the bone surface with a fine-line marker and verified using a digital caliper. The center-to-center distance between holes was maintained at ≥10 mm, and the distance from each hole center to the specimen edge was also ≥10 mm. All experiments were performed using K-wires from the same manufacturing lot. To reduce tool-related variability, a new 2.0-mm K-wire was used for every hole. Specimen number and hole capacity. In total, 11 distal porcine humeri were used. With a center-to-center spacing of ≥10 mm and a ≥ 10 mm distance from hole center to the specimen edge, the lateral condyle region could typically accommodate approximately six non-interfering holes per specimen.

### 2.3 Experimental groups

Definition of a hole block. A “hole block” was defined as a set of three non-overlapping holes on the same specimen, corresponding to the three parameter levels (one hole per level), with the execution order randomized within the block.

#### 2.3.1 Robotic consistency and robustness tests

Two repeated-measures parameter studies were performed within the robotic drill-through stopping system:

Feed-rate sensitivity test. Three specimens were used. Each specimen contributed two hole blocks (i.e., six holes per specimen). Each block included three holes corresponding to feed rates of 0.5, 1.0, and 1.5 mm·s^−^¹ (one hole per level; randomized order). This yielded 6 blocks (18 holes) and *n* = 6 hole observations per feed level.

Spindle-speed sensitivity test. Three additional specimens (no overlap with the feed-rate test) were used with the same block structure (two blocks per specimen), testing spindle speeds of 900, 1,200, and 1,500 r min^−^¹. This yielded 6 blocks (18 holes) and *n* = 6 observations per speed level. For both tests, the allocation of hole positions and the order of drilling were randomized to reduce sequence effects and location bias. Quality control measures included the use of identical consumables from the same batch, a consistent entry-angle guide for all holes, rigid clamping of specimens to avoid micromotion, and preplanned hole locations to ensure that adjacent holes did not mechanically interfere with one another.

#### 2.3.2 Robotic vs. manual drilling (paired comparison)

For the paired comparison, the robotic group used the force-sensing closed-loop stopping system, whereas the manual group was operated by a senior pediatric orthopedic surgeon with ≥10 years of clinical experience. In each pair, drilling was performed at adjacent, non-overlapping sites on the same bone, with the robotic and manual holes forming a matched pair. A paired block was defined as two adjacent, non-interfering holes on the same specimen, consisting of one robotic hole and one manual hole. Five specimens were used for the paired comparison, and each specimen contributed two paired blocks (four holes total: two robotic and two manual). These specimens were distinct from those used in the robotic parameter tests. In the robotic condition, spindle speed was set to 1,200 r min^−^¹ and feed rate was prescribed at 1.0 mm·s^−^¹. In the manual condition, the handheld drill was set to a nominal spindle speed of 1,200 r min^−^¹, while wire advancement was surgeon-controlled (unconstrained) to emulate routine clinical practice. To minimize device-related confounding, both groups used the same K-wire diameter and nominal spindle speed; however, the handheld drill differed in motor/inertia and feed was not instrumented. Therefore, the comparison primarily reflects the benefit of closed-loop stopping under typical clinical handheld conditions rather than an isolated actuator-only effect. In total, 10 paired blocks were obtained (*n* = 10), each consisting of one robotic and one manual hole. Adverse events—including K-wire jamming, overload shutdown, and obvious deviation or loss of guidance—were recorded. Drill specifications and positioning procedures were kept identical between groups.

### 2.4 Experimental protocol

For all experiments, the bone block was fixed on the platform with the drilling axis oriented 60° to the longitudinal axis of the humerus and 0° to the coronal plane. In each trial, a 2.0-mm K-wire was introduced from the lateral supracondylar region toward the medial–proximal cortex, passing through cancellous bone until breakthrough of the far cortex.

In the robotic group, drilling was performed with a UR10-based robotic system equipped with an integrated electric drill (model WS6090-48, HQuDJ, China; rated power 500 W; speed range 0–3,000 r min^−^¹). The force-sensing controller detected the characteristic drop in axial force and then automatically issued a stop command. In the manual group, drilling was performed using a conventional orthopedic power drill (model BJ1107B, Bojin, China; speed range 0–1,200 r min^−^¹). The surgeon relied on tactile and auditory cues to recognize cortical breakthrough and attempted to promptly reduce force and stop advancement to minimize inertial overdrilling.

K-wire diameter and batch were identical between groups. All experiments used 2.0 mm × 150 mm Kirschner wires (Yutong, China) from the same manufacturing lot. To minimize tool-related variability, a new K-wire was used for each hole. Before data collection, a standardized familiarization session was conducted. Any obvious deviation from the intended trajectory, overload shutdown, or other anomalies were documented and managed according to a predefined protocol. Throughout the experiments, the allocation of hole positions and the sequence of drilling were randomized, and a unified positioning procedure was applied to ensure reproducibility.

### 2.5 Robotic drilling system and force-sensing control algorithm

**Figure 1:**
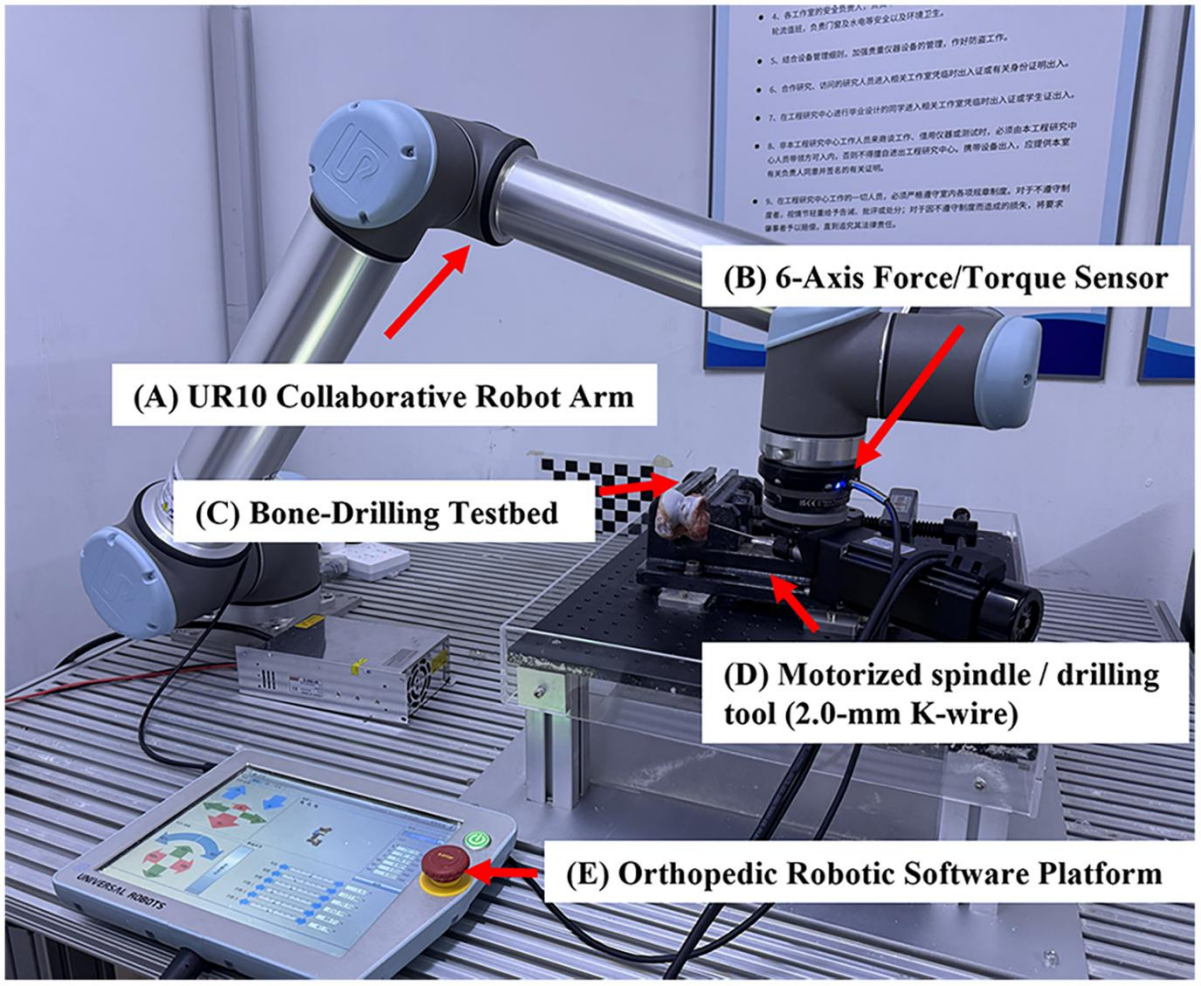
Force-sensing robotic bone drilling system. **(A)** UR10 collaborative robotic arm; **(B)** si*x*-axis force/torque sensor mounted at the end effector; **(C)** drilling platform with adjustable clamps; **(D)** electric spindle and drilling tool (2.0-mm Kirschner wire); **(E)** controller and software interface. Bone specimens are rigidly fixed to the platform. Force and torque signals are sampled at 125 Hz and time-synchronized with position data, enabling real-time detection of axial force drops and automatic execution of stop commands.

The custom force-controlled bone drilling system (Figure 1) consisted of a six-degree-of-freedom UR10 robotic arm, a si*x*-axis force/torque sensor, an electric spindle with controller, and dedicated fixation jigs. The system supported initialization of the robot and spindle, configuration of motion parameters, real-time sensor acquisition, and data logging. Force/torque data were sampled at 125 Hz and time-synchronized with position data.

Drilling was executed within a predefined safe thrust range. Robot feed was executed in a position/velocity-controlled mode with a constant commanded feed rate. Brief transient deviations in instantaneous measured velocity may occur around cortical breakthrough due to local compliance changes; however, the command profile was not intentionally altered. Drill-through stopping was triggered by a transient drop in axial force (Fx). The primary penetration criterion was defined as:

$$\Delta F_{x}=F_{x}(t_{1})-F_{x}(t_{1}-t_{2}),$$

where $F_{x}(t_{1})$ is the instantaneous axial force at time $t_{1}$, and $F_{x}(t_{1}-t_{2})$ is the axial force at an earlier time $t_{1}-t_{2}$. Penetration was flagged when all of the following conditions were simultaneously satisfied:

1. $|F_{x}(t_{1})|\geq F_{\mathrm{arm}}$ and $|F_{x}(t_{1}-t_{2})|\geq F_{\mathrm{arm}}$ (both samples above a preset arm force threshold);
2. $\Delta F_{x}\geq \theta_{F}$ (force drop exceeding a preset threshold);
3. The above condition persisted for at least *n* consecutive samples.

**Figure 2:**
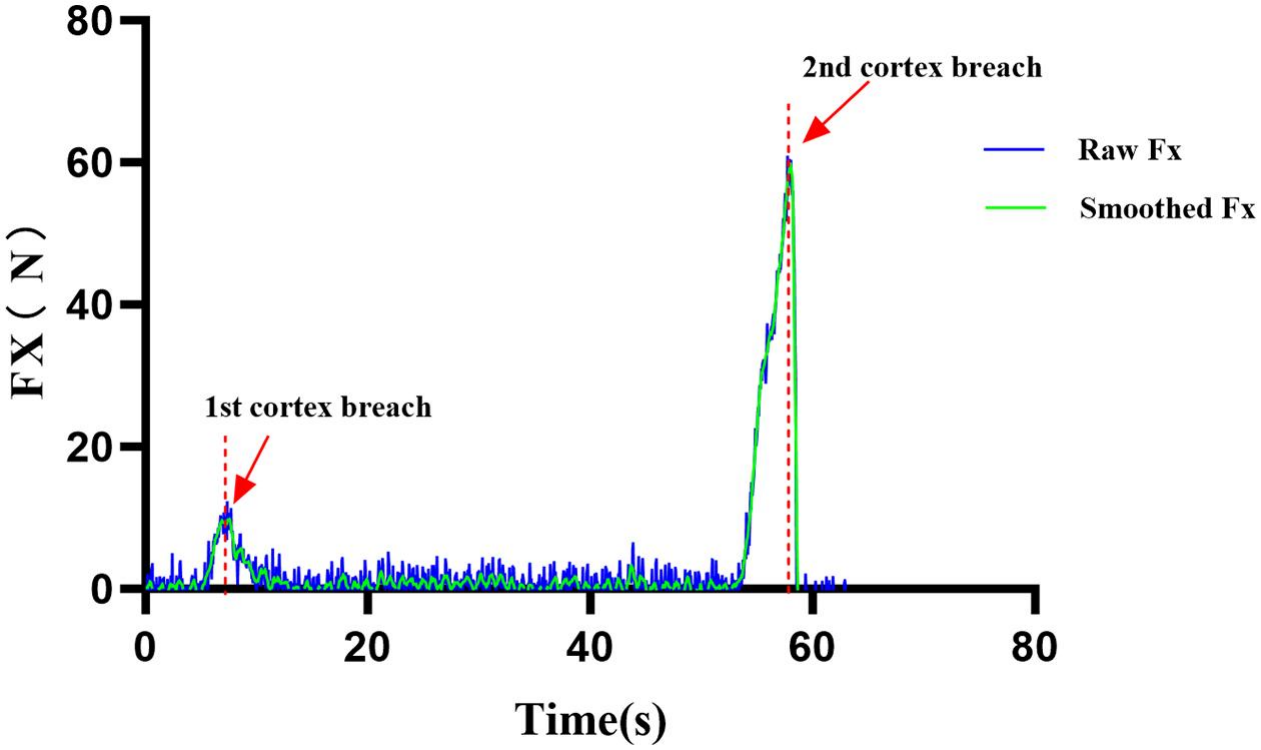
Time history of axial drilling force (Fx) with force-drop–based penetration detection.

With a sampling frequency of $f_{s}=125\;\mathrm{Hz}$, the sampling period was $T_{s}=8\;\mathrm{ms}$. Typical parameter settings were $t_{2}=L\cdot T_{s}$ (e.g., L=5), a force-drop threshold $\theta_{F}=5\;N$, an arm-force threshold $F_{\mathrm{arm}}=10\;N$, and n=3 consecutive samples. Once the penetration criterion was met, the controller immediately terminated feed motion and issued a stop command to the spindle (Figure 2). Before formal testing, the end-to-end latency from force-drop detection to complete spindle stop was measured experimentally to confirm that the system performance was reproducible under the test conditions.

Raw force/torque signals were denoised using low-pass and median filtering before being passed to the penetration detection module for analysis.

### 2.6 Fail-safe and safety constraints

To mitigate potential failure modes (e.g., sensor artifacts, missed detection, and actuator non-response), the system incorporated multi-layer safety constraints. First, axial force was monitored in real time (with torque recorded for reference); exceeding predefined hard limits (Fx > Fmax) immediately halted feed motion and commanded spindle stop. Hard limits were set *a priori* based on pilot testing to remain below levels associated with wire bending, bone cracking, or fixture slippage. Second, travel/time limits were applied to prevent uncontrolled advancement if breakthrough was missed. For each planned trajectory, the maximum allowed travel was defined as Lmax = Lbone + ΔL (ΔL = 5 mm), and the maximum allowed drilling time was tmax = Lmax/vfeed + Δ*t* (Δ*t* = 5 s), where vfeed is the prescribed feed rate. Lbone was measured for each planned hole as the trajectory length from the outer surface of the near cortex to the outer surface of the far cortex along the drilling axis. If the breakthrough criterion was not met within either limit, the controller halted feed motion and commanded spindle stop as a fail-safe. Hardware and software emergency stops were available throughout, and the operator could manually override the system at any time. All safety events (trigger type, timestamp, and force/position, torque recorded for reference) were logged for traceability.

### 2.7 Outcome measures and calibration

The primary outcome measure was post-penetration overdrill depth (mm). Using the outer surface of the far cortex as the reference endpoint, overdrill depth was calculated as the difference between (1) the depth from the outer surface of the near cortex at the entry point to the deepest point of the drill channel and (2) the actual cortical thickness, defined as the distance between the outer surfaces of the near and far cortices along the same drilling axis. Measurements were obtained with a vernier caliper (resolution 0.01 mm) and recorded to 0.01 mm. Each hole was measured twice, and the mean of the two readings was used. If burrs or collapse were present at the channel edge, the entrance was gently cleaned with a deburring tool before measurement.

To assess measurement reliability, two investigators independently measured each hole. If the absolute difference between their readings exceeded 0.20 mm, a third measurement was performed, and the final value was taken as the mean of all three measurements. Before each testing day, the depth rod of the caliper was calibrated to zero and across a reference span using gauge blocks, with an acceptance tolerance of ±0.02 mm. All measurements were performed in a temperature-controlled laboratory at 24 °C.

Adverse events were recorded for each hole according to predefined categories: K-wire jamming (requiring reverse withdrawal), overload shutdown (device protection triggered), obvious deviation from the intended trajectory or loss of guidance (drilling axis deviating from the planned angle), and measurement interference (e.g., entrance collapse affecting depth readings). The corresponding management and whether the hole was included in the final analysis were documented.

### 2.8 Statistical analysis

The primary outcome variable was post-penetration overdrill depth (mm). For the parameter sensitivity tests (repeated measures), a one-factor, three-level design (k = 3; factor: spindle speed or feed rate) was used. Within-subject residuals were first assessed for normality using the Shapiro–Wilk test and inspection of Q–Q plots. When assumptions were met, one-way repeated-measures ANOVA was applied, followed by Tukey *post hoc* comparisons. If variance or sphericity assumptions were violated, Greenhouse–Geisser (G–G) correction was used. In cases of markedly non-normal distributions or clear outliers, the Friedman test was used instead, with Dunn's multiple comparisons and Holm adjustment for pairwise tests. For the main tests, F statistics $F(df_{1},df_{2})$, *p* values, and partial $\eta^{2}$ were reported; for pairwise comparisons, mean differences (mm), 95% confidence intervals (CI), and adjusted *p* values (p_adj) were presented.

For the paired comparison between the robotic and manual groups, adjacent hole sites on the same bone formed a paired block. Within each block, one robotic value ($R_{i}$) and one manual value ($M_{i}$) were obtained, and the paired difference was defined as $D_{i}=M_{i}-R_{i}$. A two-sided paired t-test (*α* = 0.05) was used to compare groups, with mean difference, 95% CI, *p* value, and effect size (Cohen's d_z) reported. The normality of $D_{i}$ was evaluated with the Shapiro–Wilk test; if the distribution deviated from normality, a Wilcoxon signed-rank test was additionally performed. All analyses were conducted using GraphPad Prism version 10.12. All statistical tests were two-sided, with a significance level of *α* = 0.05.

### 2.9 Sample size estimation

For the repeated-measures parameter tests (one factor, three levels), sample size was estimated based on a pilot standard deviation of paired differences of *σ*_d = 0.20 mm and a minimal clinically important difference (MCID) of Δ = 0.30 mm, yielding an effect size of $d=\Delta /\sigma_{d}=1.5$. With *α* = 0.05 and powe*r* = 0.80, G*Power estimated that *n* = 5 paired blocks were required. To account for potential data loss or exclusions, the planned sample size was increased to *n* = 6 blocks (18 holes per experiment).

For the robotic vs. manual paired comparison, a pilot series of six pairs yielded a mean paired difference and a standard deviation $SD_{D}=0.63\;\mathrm{mm}$, corresponding to an effect size of . With *α* = 0.05 and power = 0.80, G*Power indicated that a minimum of *n* = 3 pairs would be sufficient. Given the likelihood that the pilot effect size was overestimated and to allow for potential exclusions due to adverse events, the target sample size was increased to *n* = 10 pairs (20 holes in total).

Blue curve: raw Fx; green curve: filtered Fx. Between 5 and 7 s, initial contact with the thin near cortex (orange arrow) causes a rise in Fx, followed by a mildly fluctuating steady cutting phase. Around 53–54 s, Fx increases again, indicating approach to the far cortex. At 58–59 s, cortical breakthrough occurs (red arrow), producing an abrupt drop in Fx that satisfies the force-drop criterion and triggers the control algorithm to terminate feed motion and stop the spindle, thereby limiting overdrilling. Horizontal axis: time (s); vertical axis: Fx (N).

Post-penetration overdrill depth at feed rates of 0.5, 1.0, and 1.5 mm·s^−^¹ (*n* = 6 hole blocks derived from 3 specimens; each specimen contributed two blocks, and each block contributed one hole per level; total 18 holes). Dots represent individual holes; Bars indicate group means, and error bars represent standard deviations (SD). Data were analyzed with one-way repeated-measures ANOVA (Geisser–Greenhouse correction where appropriate), followed by Tukey *post hoc* pairwise comparisons. A *p* value < 0.05 was considered statistically significant; “ns” denotes non-significant pairwise differences.

Distribution of post-penetration overdrill depth at spindle speeds of 900, 1,200, and 1,500 r·min^−^¹ ((*n* = 6 hole blocks derived from 3 specimens; each specimen contributed two blocks, and each block contributed one hole per level; total 18 holes). Dots represent holes; lines (if shown) connect repeated measures within the same specimen. bars indicate group means, and error bars represent standard deviations (SD). Brackets indicate pairwise Tukey *post hoc* comparisons and their significance. Statistical analysis was performed using one-way repeated-measures ANOVA (with Geisser–Greenhouse correction where appropriate). A *p* value < 0.05 was considered statistically significant; “ns” indicates non-significant pairwise differences.

## Results

### 3.1 Feed-rate sensitivity test (0.5/1.0/1.5 mm·s^−^¹)

**Figure 3:**
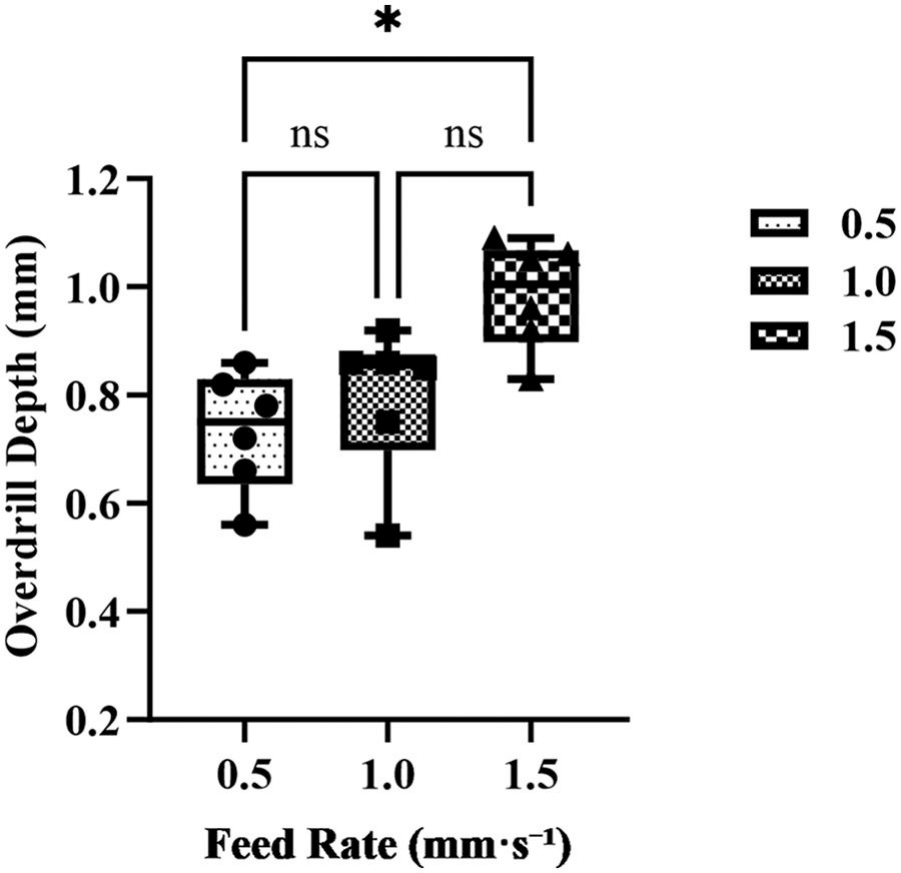
Effect of feed rate on post-penetration overdrill depth.

The feed-rate test included 3 specimens and 6 hole blocks (18 holes; *n* = 6 per level). A one-way repeated-measures ANOVA (Geisser–Greenhouse corrected) showed a significant main effect of feed rate on overdrill depth, *F*(1.992, 9.958) = 6.382, *p* = 0.016, with a large effect size (partial *η*^2^ = 0.56). The mean  ± SD overdrill depths were 0.733 ± 0.111 mm at 0.5 mm·s^−^¹, 0.797 ± 0.137 mm at 1.0 mm·s^−^¹, and 0.985 ± 0.100 mm at 1.5 mm·s^−^¹. Tukey *post hoc* comparisons showed that 1.5 mm·s^−^¹ produced significantly greater overdrilling than 0.5 mm·s^−^¹ (mean difference +0.252 mm; 95% CI, 0.0065–0.497; p_adj = 0.0456). The differences between 0.5 and 1.0 mm·s^−^¹ (mean difference −0.063 mm; 95% CI, −0.3017–0.1750; p_adj = 0.6832) and between 1.0 and 1.5 mm·s^−^¹ (mean difference −0.188 mm; 95% CI, −0.4200–0.04335; p_adj = 0.0981) were not statistically significant (Figure 3).

### 3.2 Spindle-speed sensitivity test (900/1,200/1,500 r min^−^¹)

The spindle-speed test included 3 specimens and 6 hole blocks (18 holes; *n* = 6 per level). One-way repeated-measures ANOVA with Geisser–Greenhouse correction (*ε* = 0.6786) indicated no significant main effect of spindle speed on overdrill depth, F(1.357, 6.786) = 0.0459, *p* = 0.8998, with a negligible effect size (partial *η*^2^ ≈ 0.009). The mean ± SD overdrill depths were 0.8217 ± 0.0962 mm at 900 r min^−^¹, 0.8200 ± 0.1455 mm at 1,200 r min^−^¹, and 0.8050 ± 0.1297 mm at 1,500 r min^−^¹. None of the Tukey *post hoc* pairwise comparisons reached significance:

900 vs. 1,200 r min^−^¹, Δ = 0.0017 mm (95% CI, −0.1641–0.1675; p_adj = 0.9994);

900 vs. 1,500 r min^−^¹, Δ = 0.0167 mm (95% CI, −0.1370–0.1704; p_adj = 0.9345);

1,200 vs. 1,500 r min^−^¹, *Δ* = 0.0150 mm (95% CI, −0.2410–0.2710; p_adj = 0.9802).

**Figure 4:**
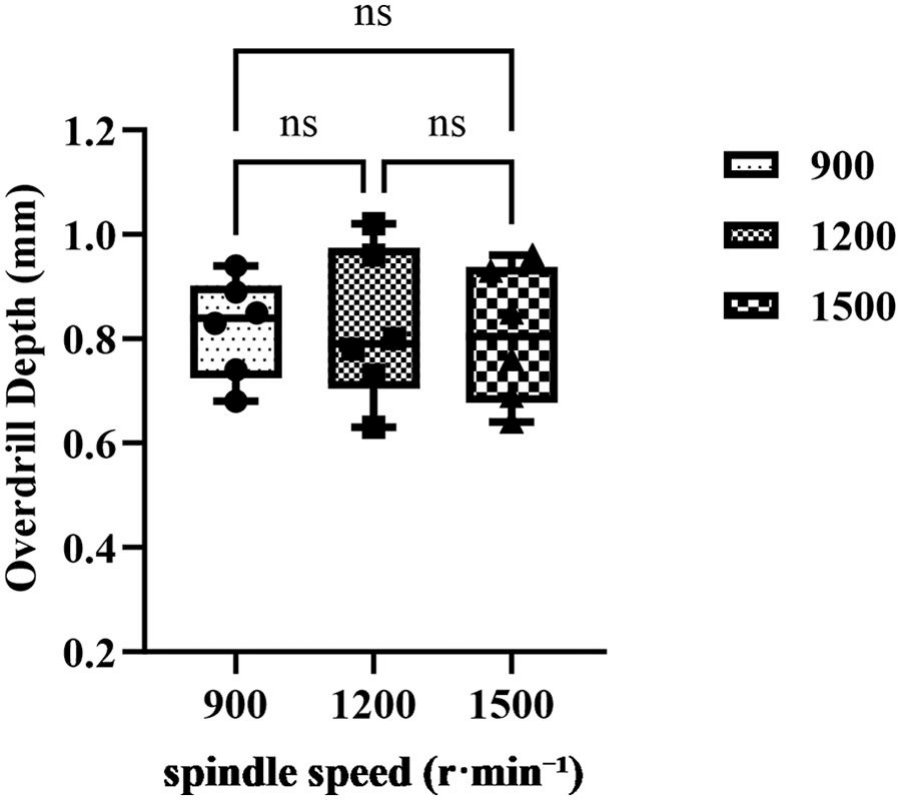
Effect of spindle speed on post-penetration overdrill depth.

These findings indicate that, within the tested range, overdrill depth was essentially insensitive to spindle speed (Figure 4).

### 3.3 Paired comparison between robotic system and manual drilling

**Figure 5:**
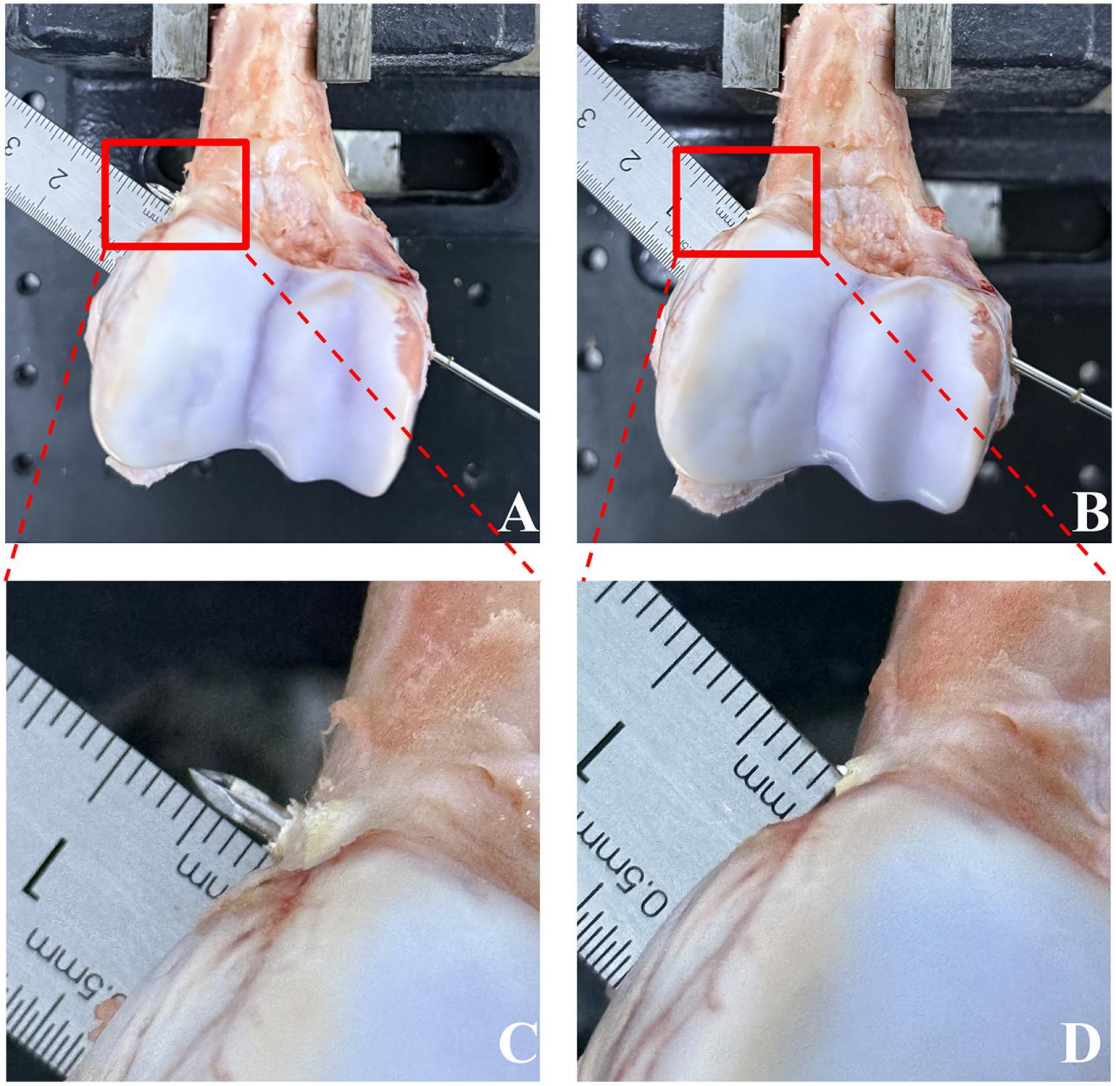
Comparison of overdrilling with manual drilling vs. force-sensing robotic drill-through stopping **(A–D)**. **(A)** Gross view of a representative porcine distal humerus specimen after manual K-wire drilling. The red dashed box marks the area magnified in **(C)**, where the K-wire tip protrudes beyond the outer surface of the far cortex, with an overdrill distance of approximately 7 mm. **(B)** Gross view of a representative specimen after force-sensing robotic drill-through stopping. The corresponding magnified view in **(D)** shows the K-wire tip terminating within about 1 mm beyond the outer surface of the far cortex, illustrating the system's control of post-penetration overdrilling.

The paired comparison included 5 specimens and 10 paired blocks (20 holes). Compared with manual drilling at adjacent sites on the same bone, the force-sensing robotic system substantially reduced post-penetration overdrill depth. Mean overdrill depth was 0.865 ± 0.121 mm in the robotic group and 6.595 ± 1.525 mm in the manual group. The paired mean difference (Manual−Robot) was 5.730 mm (95% CI, 4.665–6.795). A paired t-test showed a highly significant difference, *t*(9) = 12.17, *p* < 0.0001, with a very large effect size (Cohen's d_z = 3.85; partial *η*^2^ = 0.943), indicating a marked and highly consistent advantage of the robotic system (Tables 1, 2, Figure 5). In practical terms, overdrilling was controlled to <1 mm with the robotic system, whereas manual drilling resulted in overdrill depths of approximately 6.6 mm (Figure 5).

**Table 1:** Descriptive statistics of post-penetration overdrill depth (mm).

Group	*n*	Mean ± SD (mm)	Median [IQR] (mm)	Min–Max (mm)
Robot	10	0.865 ± 0.121	0.835 [0.775–0.955]	0.690–1.080
Manual (senior)	10	6.595 ± 1.525	6.565 [5.420–7.790]	3.560–8.650

Adjacent holes on the same bone were treated as paired units (*n* = 10 pairs). IQR, interquartile range.

**Table 2:** Paired comparison of post-penetration overdrill depth (manual−robot; mm).

*n*(pairs)	Mean difference (mm)	SD of differences (mm)	95% CI (mm)	*t*(df)	*p* value	Cohen's d_z
10	5.730	1.489	4.665–6.795	12.17	<0.0001	3.85

Two-sided paired t-test; *α* = 0.05. Cohen's d_z = *t*/√*n*.

### 3.4 Adverse events

No K-wire jamming, overload-related shutdown, or noticeable trajectory deviation/loss of guidance was observed in either group. No fail-safe events (force/torque limit stop, travel/time-limit stop, or emergency-stop activation) occurred. No holes were excluded from the analysis due to adverse events.

## 4 Discussion

In closed percutaneous K-wire fixation of pediatric supracondylar humeral fractures, pin trajectories are placed within an anatomical corridor adjacent to major neurovascular structures, and neurovascular compromise is a well-recognized concern in this injury setting (3, 4, 8, 13). Operative technique guidance emphasizes engaging the far cortex while keeping pin-tip protrusion beyond the outer cortex as small as possible, with fluoroscopic confirmation used to assess the extent of protrusion (14). In addition, nerve-protection–oriented technical modifications have been proposed that explicitly highlight intraoperative protection strategies and recommend minimizing far-cortex protrusion (e.g., ≤2 mm on fluoroscopy) (15).

The anatomic margin can be only a few millimeters. Pediatric MRI-based analyses have shown that the radial nerve can approach the lateral distal humeral cortex within only a few millimeters in some segments (16). Cadaveric/anatomic evidence further supports substantial variability and a non-negligible risk of iatrogenic nerve injury during percutaneous pin/half-pin instrumentation around the distal humerus (17). Although vascular complications after pinning are uncommon, brachial artery pseudoaneurysm associated with K-wire use has been reported (18, 19), illustrating that even a few millimeters of unrecognized protrusion beyond the far cortex can be clinically consequential in the compact antecubital soft-tissue envelope.

In routine practice, depth control remains challenging because surgeons typically rely on intermittent fluoroscopy to judge both trajectory and the extent of pin protrusion beyond the far cortex. However, fluoroscopic assessment may underestimate true protrusion: in a synthetic bone model simulating pediatric supracondylar humerus and distal radius pinning, fluoroscopic measurements underestimated true pin protrusion by approximately 1.5 mm on average (20). This matters because a pin that appears to protrude only slightly on fluoroscopy may, in reality, exceed commonly recommended minimal-protrusion margins (e.g., ≤2–3 mm) (14, 15, 20). Related work on bicortical drilling has shown that plunge/overshoot can occur with handheld techniques, and controlled-advancement drills have been proposed to reduce it (21). In this context, our *ex vivo* results—showing that the force-sensing closed-loop system limited post-penetration overdrill to ∼1 mm—suggest drill-through detection with automatic stopping as an engineering adjunct to fluoroscopic checks, particularly at cortical breakthrough when tactile cues and reaction delay are most limiting.

When relying on tactile and auditory cues intraoperatively, human reaction delay is unavoidable. Reaction times for trained surgeons have been reported in the range of 313–358 ms (22). During this interval, the power drill continues to deliver torque and the K-wire advances with residual kinetic energy. Because of the combined rotational and translational inertia, the wire continues to move forward even after the surgeon “feels a give” resulting in several millimeters of inertial overshoot. In addition, the mechanical signature of cortex perforation is influenced by bone quality, entry angle, boundary support, and soft-tissue clamping. Variations in these factors may attenuate or distort force and sound cues, causing them to be delayed, or masked by noise and thereby increasing the uncertainty of manual stopping.

To mitigate these issues, we used filtering, a short-window force-change metric, and a consecutive-sample rule to reduce noise-driven triggers. Axial force is streamed in real time, and breakthrough is identified when the force drop exceeds predefined thresholds and persists across several successive samples, which helps reduce false triggers from transient noise. We also implemented conservative safety constraints, including a thrust-force limit and predefined fail-safe stop conditions. Unlike manual stopping, the control decision is driven by the real-time signal and executed within a millisecond-scale control loop. For context, published acoustic-based methods report detection-to-trigger delays of ∼64–139 ms (23). In our setup, the measured end-to-end latency—from detection to complete spindle stop—was 50–100 ms, thereby shortening the breakthrough-to-stop interval and reducing overshoot due to reaction delay and system inertia.

Previous experimental and review studies have shown that drilling thrust force is strongly affected by feed rate and spindle speed (24). Higher feed rates generally increase thrust, whereas the effect of spindle speed is more complex: at low speeds, thrust tends to be higher, and moderate increases in speed may reduce thrust, but the overall pattern depends on drill geometry and material. Generally, feed rate exerts a stronger influence than speed. Parameter studies of K-wire drilling have likewise suggested that both speed and feed affect force levels (25). In our tests, raising the feed rate from 0.5 to 1.5 mm·s^−^¹ produced a small but measurable increase in mean overdrill depth (0.73–0.98 mm), consistent with greater thrust and momentum increasing residual advance during the breakthrough-to-stop interval. Importantly, overdrill remained <1 mm across all feed settings, suggesting that the controller tolerated feed variation within the tested range. By contrast, overdrill did not differ detectably across 900–1,500 r min^−^¹. This likely reflects that, under our specific setup (material, 2.0-mm K-wire, and algorithm thresholds), trigger timing was governed primarily by the force-drop event at breakthrough, and any speed-related change in cutting efficiency had only a minor influence on when the criterion was met.

The present study demonstrated the practical benefit of force-sensing closed-loop control. The system consistently limited post-penetration overdrill depth to approximately 1 mm, which was markedly better than manual drilling (robotic 0.865 ± 0.121 mm vs. manual 6.595 ± 1.525 mm; paired mean difference 5.730 mm). Mechanistically, this advantage arises from the continuous rise in axial force as the far cortex is approached, followed by a sharp drop at breakthrough. Once the force-drop criterion is satisfied, the controller immediately terminates feed motion and stops the spindle, minimizing residual displacement after perforation and achieving a reproducible “breakthrough-and-stop” behavior. Such millisecond-level sensing–actuation loops operate faster than human tactile and auditory response and can be reproduced reliably under the same setup and drilling posture.

Our human-vs.-robot experiment was meant to anchor the engineering result to the clinical baseline—how an experienced surgeon drills by hand—rather than to position the system against every possible auto-stop method. Using the same *ex vivo* model and a paired setup, we could quantify the added value of closed-loop stopping specifically at far-cortex breakthrough. Comparisons with other automatic shutdown strategies (acoustic, torque, displacement, or controlled-advancement drills) are a logical next step and will require implementation and tuning under identical conditions; we plan to address this in future studies.

From a clinical perspective, compressing overdrill depth to around 1 mm not only reduces the probability of inadvertently entering a neurovascular channel but may also offer a range of additional benefits for both patients and surgeons. Immediate stopping at breakthrough is expected to prevent the K-wire tip from “sweeping” through soft tissues outside the bone, potentially reducing the risk of muscle interspace contusion, hematoma formation, and repeated passes due to suboptimal trajectories. A higher first-pass success rate and fewer corrective attempts may, in turn, shorten operative and anesthesia time, which is particularly relevant in younger children with increased airway management and recovery risks. Under a “perforate–stop–confirm” workflow, the surgeon's uncertainty about whether the cortex has been breached and whether the tip has travelled beyond safe limits may be reduced, which in theory could decrease the number of fluoroscopy checks and overall radiation exposure. By turning the ideal maneuver of “stopping 1–2 mm beyond the cortex” from a skill heavily dependent on experience into a repeatable, parameterized process, closed-loop stopping may also flatten the learning curve for less-experienced surgeons and improve consistency across teams.

This study has several limitations. First, we used distal porcine humeri as a surrogate for the pediatric elbow environment. Although this model offers good controllability and repeatability, differences remain compared with pediatric bone in cortical thickness, trabecular architecture, and hydration state, which may affect the absolute force/torque levels. Second, in the handheld condition, actual spindle speed and axial hand pressure were not instrumented and therefore could vary across trials; this was intended to reflect clinical realism but remains a limitation. Future work will test thinner/softer bone conditions and will quantify actual spindle speed and feed/advancement variability in the handheld setting. In addition, we focused on post-penetration overdrill depth as the primary safety endpoint and did not simultaneously quantify other potentially relevant outcomes (e.g., temperature elevation, microcracking at the cortical edge, or trajectory deviation). Future studies may incorporate additional measurements (e.g., thermal monitoring and geometric/pose tracking) to more comprehensively characterize drilling performance.

Finally, under boundary conditions such as extremely thin cortex, insufficient clamping, interference from neighboring holes, or progressive K-wire wear, the magnitude and slope of the force-drop signal may be reduced, potentially making detection more challenging. To improve robustness under these conditions, future systems may benefit from higher sampling frequencies and faster actuators, as well as multimodal feature fusion—for example, combining force cues with torque, acoustic, or displacement signals—to improve penetration detection reliability in more diverse clinical scenarios. In addition, fair benchmarking against other automatic shutdown strategies (e.g., acoustic- or motor-current–based triggers, or alternative force-based controllers) would require implementation and tuning on the same platform under identical conditions; this will be examined in future studies.

## 5 Conclusions

In an *ex vivo* porcine distal humerus model simulating K-wire advancement and far-cortex breakthrough in pediatric supracondylar humeral fracture pinning scenarios, a force-sensing closed-loop drill-through stopping system consistently limited post-penetration overdrill depth to ∼1 mm. Across parameter tests, increasing feed rate from 0.5 to 1.5 mm·s^−^¹ produced only a modest increase in overdrilling (0.73–0.98 mm), while spindle speed between 900 and 1,500 r min^−^¹ had no detectable effect. In paired tests against a senior surgeon, the system markedly reduced overdrill depth (0.87 ± 0.12 mm vs. 6.60 ± 1.53 mm), with a mean paired difference of 5.73 mm (Manual—Robot) and an ∼87% relative reduction. Overall, the system demonstrated robust performance within clinically relevant settings and may increase the safety margin after far-cortex breakthrough by limiting overdrilling. Whether these reductions translate into fewer neurovascular events or fewer corrective maneuvers requires validation in clinically representative models and prospective clinical studies.

## Data Availability

The raw data supporting the conclusions of this article will be made available by the authors, without undue reservation.
